# A Correlative Classification Study of Schizophrenic Patients with Results of Clinical Evaluation and Structural Magnetic Resonance Images

**DOI:** 10.1155/2016/7849526

**Published:** 2016-10-24

**Authors:** Wen-Lin Chu, Min-Wei Huang, Bo-Lin Jian, Chih-Yao Hsu, Kuo-Sheng Cheng

**Affiliations:** ^1^Institute of Biomedical Engineering, National Cheng Kung University, Tainan 701, Taiwan; ^2^Department of Psychiatry, Taichung Veterans General Hospital, Chiayi Branch, Chiayi 600, Taiwan; ^3^Department of Aeronautics and Astronautics, National Cheng Kung University, Tainan 701, Taiwan

## Abstract

Patients with schizophrenia suffer from symptoms such as hallucination and delusion. There are currently a number of publications that discuss the treatment, diagnosis, prognosis, and damage in schizophrenia. This study utilized joint independent component analysis to process the images of GMV and WMV and incorporated the Wisconsin card sorting test (WCST) and the positive and negative syndrome scale (PANSS) to examine the correlation of obtained brain characteristics. We also used PANSS score to classify schizophrenic patients into acute and subacute cases, to analyze the brain structure differences. Finally, we used brain structure images and the error rate of the WCST as eigenvalues in support vector machine learning and classification. The results of this study showed that the frontal and temporal lobes of a normal brain are more apparent than those of a schizophrenia brain. The highest level of classification recognition reached 91.575%, indicating that the WCST error rate and characteristic changes in brain structure volume can be used to effectively distinguish schizophrenia and normal brains. Similarly, this result confirmed that the WCST and brain structure volume are correlated with the differences between schizophrenia and normal participants.

## 1. Introduction

Typical schizophrenia tends to occur in young adults, in whom the disability can last for life, and obstruct social functions such as personal relationship, job, and self-care. The social function impairments of patients with late-stage schizophrenia can result in substantial cost burden to the patients, their families, and the healthcare system [1, 2]. In the past, many research articles described studies on schizophrenia prognosis and involved a wide range of aspects. However, the results of these studies showed diversity. In addition, the methodology of these studies suffered from issues such as definition of patients, consistency of description and measurement tools, sampling, and study design. The standardization of diagnosis interview and symptom measurement tools has improved the reliability and comparability of studies on prognosis and helped accumulate knowledge related to schizophrenia. However, the heterogeneity of schizophrenia remains a major difficulty in studies on this condition. Current clinical description and definition of this condition normally involve interview as the diagnosis method and clinical tools that evaluate the expression of human cognitive function, such as the WCST, PANSS, and Continuous Performance Test (CPT). Liu et al. conducted a correlation analysis of the results from the PANSS and CPT and found that the presence of only negative symptoms was significantly correlated with psychological variables that measure attention and alertness [3]. When WCST was first developed, it was expected to be able to evaluate the executive function of participants, including abstract reasoning ability and resolution strategies to achieve goals, and the ability to modify the types of cognition and adjust impulse responses according to environmental feedback [4–6]. It can also test working memory, which is controlled by the dorsal-lateral prefrontal system [7]. The WCST consists of four stimulus cards and 128 response cards, and the result contains nine scores [4]. Some studies suggested that the result of the WCST could be represented with three scores [8, 9]. For example, Bell et al. [8] believed that the WCST result can be represented by perseveration, nonperseveration error, and inefficient sorting, while Koren et al. [9] believed that it can be represented by perseveration, failure to maintain set, and idiosyncratic sorting.

In the earlier days, Brief Psychiatric Rating Scale was widely used for the evaluation of psychological symptoms and their severity [10]. In recent years, the application of PANSS has gradually increased for the evaluation of psychological symptoms and their severity in patients with schizophrenia [11–15]. Kay and Sevy [16] utilized factor analysis and identified five independent factors in the PANSS, among which the factors of damage in the cognitive function originate from the lack of organization of thinking in the active symptom, as well as difficulties in abstract thinking in the negative symptom. These studies demonstrate that improving the effective diagnosis of schizophrenia in patients is very important.

In 1976, Johnstone et al. [17] discovered with CT that the pathogenesis of schizophrenia was possibly correlated to damage of brain structure. Other related studies also showed that brain structure GMV reduction in the left Heschl gyrus and left superior temporal gyrus is significantly different in patients with schizophrenia [18]. Observation with diffusion MRI showed that the white matter and grey matter structures were significantly different between patients with schizophrenia and normal participants [19]. In addition, studies comparing the brains with first-episode psychosis and normal brains demonstrated a reduction in the right frontal WMV in early-stage schizophrenia [20]. It is apparent from the literature that there are differences between the changes in brain volume structure of schizophrenia and normal participants.

T1-weighted images are a type of high-resolution three-dimensional images. In this study, we used VBM for accurate observation of changes in brain tissue morphology [21–23]. Sarró et al. [24] used voxel-based structural imaging to observe the variation in the GMV with or without dyskinesia in schizophrenia brains. Apart from VBM, ICA is a multivariate analysis technique that separates complicated data or signals into statistically independent non-Gaussian signals. Because it is currently not possible to build completely correct neurological model based on MR images, analysis based on Generalized Linear Model (GLM) is mostly used [24–26]. The application of ICA to MR images provides another possible data analysis method and can utilize more information than GLM, to identify structural information in the data. In particular, functional MRI (fMRI) has been widely used in research [27, 28]. This study utilized jICA, which inherits the strengths of ICA, and it can observe the common information among multiple data types [29]. Moosmann et al. [30] adopted the jICA method to evaluate all available electrophysiological and hemodynamic information from electroencephalography and blood oxygen level dependent-fMRI. In addition, Calhoun et al. [31] studied the different stimulation in patient with schizophrenia and normal participants when attempting various tasks, using jICA to observe the fMRI data, and proposed that jICA is a simple method that can connect multiple data types while the analysis result was meaningful [32–34].

In order to discuss whether the changes in brain structure volume obtained by the WCST are correlated to the differences between patients with schizophrenia and normal participants, we adopted the classification algorithms for learning and classification in this study. If the classification recognition rate based on these eigenvalues was good, then these eigenvalues strongly correlated to the classification of patients with schizophrenia and normal participants. It is also possible to build models for predicting unknown classification and to use the result in research. The currently known classification algorithms include Neural Network, support vector machine (SVM), and Learning Vector Quantization. The primary strength of SVM is that, with relatively small sample size, it has great classification ability in terms of identification in high-dimensional space and relatively accurate classification. SVM is a learning algorithm based on statistical theories. It was first proposed by Vapnik and can be applied to the classification of linear and nonlinear data [35]. In brief, the algorithm finds a hyperplane in the space, to search for the optimal boundary of the two types of training samples, and by applying appropriate kernel functions to model complicated nonlinear boundary, to maximize the Margin distance between the two types [36]. Therefore, apart from investigating the correlation between the variation in schizophrenia and normal brains and clinical scoring in this study, we also used SVM in learning and classification to verify the correlation.

## 2. Materials and Methods

In this study, we investigated the difference in brain structure between normal subjects and patients with schizophrenia using T1-weighted images and analyzed the differences with voxel-based morphometry (VBM) and joint independent component analysis (jICA). The WCST error rate and results of data analysis such as eigenvalues were applied to the SVM classification.

### 2.1. Participants

The study included 19 schizophrenic patients (mean age: 41.6 ± 4.9 years; range: 34–57 years; 9 men, 10 women) and 16 normal control subjects (mean age: 46.9 ± 12.8 years; range: 25–64 years; 7 men, 9 women) (Table 1). The 19 schizophrenic patients had a definite clinical diagnosis of schizophrenia according to Diagnostic and Statistical Manual of Mental Disorders, Fourth Edition (DSM-IV) criteria. The patients diagnosed as a case of chronic or acute dementia according to DSM-IV criteria were excluded from the study. The 16 normal control subjects had no history of psychiatric disease, neurological disease, or drug abuse. There were no differences in age and sex. The participants were enrolled from the Department of Psychiatry, Chiayi and Wanqiao Branch, Taichung Veterans General Hospital, Chiayi, Taiwan. All participants received screening that included their medical and psychiatric history, laboratory testing, drug screening, physical examination, and structural MRI. Psychiatric diagnosis of schizophrenia was accepted using the Structured Clinical Interview from the DSM-IV and a semistructured interview conducted by a study psychiatrist [37]. After getting a complete explanation of the study procedures, all participants provided written informed consent as approved by the institutional review board. This study was approved by the ethics committee of Taichung Veterans General Hospital and conducted in accordance with Good Clinical Practice procedures and the current revision of the Declaration of Helsinki [38, 39]. The neuropsychiatric symptoms of schizophrenia were evaluated by 30-item PANSS, which provides a total score (sum of the scores of all 30 items). Each scale is rated from 1 (absent) to 7 (extreme). The PANSS assessment was performed by a qualified rater defined as a trained clinician. If possible, for a given subject, the same rater appraised this scale at all visits. Subjects were interviewed at each visit to assess the psychiatric symptoms of schizophrenia [40]. A computerized version of the WCST was applied to each participant, who was guided to match a “response” card to 1 of the 4 “stimulus” cards on the basis of 3 dimensions (color, form, or number) by pressing 1 of the 1–4 number keys on the computer keyboard [41–43]. Subjects neither were informed of the correct sorting principle nor were told when the principle would shift during the test, but they were given feedback (“Right” or “Wrong”) on the screen after each trial. The testing continued until all 128 cards were sorted.

### 2.2. Imaging Parameters

The morphological sequence we use is T1-weighted image. All participants received a whole-brain MR scan (GE Medical Systems Signa HDx) with the following image parameters: field strength = 1.5 T, repetition time (TR) = 10.428 ms, echo time (TE) = 3.128 ms, inversion time (TI) = 400 ms, slice thickness = 1.5 mm, matrix = 256 × 256, voxel size = 1.5 × 1.5 × 1.5 mm^3^, and number of slices = 120.

### 2.3. Data Analysis

*Images Preprocessing*. VBM was used for the pretreatment of MRI. As an analysis technique of neurological images, VBM is widely used for the detection of changes in the brain GMV and WMV. Every image was processed using the default settings for the VBM8 Toolbox (http://dbm.neuro.uni-jena.de/) for the MATLAB (MathWorks Inc., Sherborn, MA, USA) Statistical Parametric Mapping 8 (SPM8, Wellcome Department of Cognitive Neurology, UK; see http://www.fil.ion.ucl.ac.uk/spm/). First, the anterior commissure was set as the center of all T1-weighted images. The voxel intensities in the images were plotted as a histogram. Mixture Gaussian filter removed the curve that could affect the intensity, to avoid the issue of partial volume. These steps segmented T1-weighted images into GMV, WMV, and cerebrospinal fluid (CSF). In addition, the affine registration was carried out to the tissue probability maps during native space tissue segmentation and was aligned to 1 × 1 × 1 mm^2^ standard anatomical space coordinate axis provided by MNI 152. By using SPM8 DARTEL Toolbox, the images at the same location were aligned to known anatomical coordinate axes [44]. Finally, each image underwent statistical analysis voxel by voxel.

*Joint Independent Component Analysis*. Fusion ICA Toolbox (FIT) in MATLAB (http://mialab.mrn.org/software/fit/) was used in this study [45]. All images were converged to one-dimension vectors, the arrangement of which is shown in Figure 7. The GMV and WMV were shown in Feature 1 and Feature 2. All input parameters were reduced by Infomax algorithm, making the output joint-sources minimum mutual information [46]. These common sources were independent of each other. Mixing matrix statistical analysis was used to identify significant differences between groups.

*Visualization*. Each joint-source was converted into *Z*-score (unit standard deviation) and into three-dimensional images. The illustration was then converted into MNI brain model to be displayed in the normalized coordinate space, with threshold of *Z* > 3.5. The significant areas in all joint-sources were converted into standard Talairach space from the MNI normalized template.

### 2.4. Statistical Analysis

The statistical software used in this study was SPSS. First, age and sex covariances of patients with schizophrenia and normal controls were controlled. Then, VBM was used to carry out subsequent brain structure analysis. Because the observed voxels did not independently exist in neighboring voxels, *p* < 0.05 familywise error rate threshold was used for adjustment. Two-tailed *t*-test was used for jICA, to verify the significant differences between patients with schizophrenia and normal participants.

### 2.5. Classification SVM

SVM is a type of supervised learning structure and processes data for classification. In brief, SVM builds a hyperplane and moves it, until an appropriate boundary is found. Apart from keeping the correct classification of the hyperplane, SVM also maximizes the empty space on the side of the hyperplane [47]. The LIBSVM tools [48] are used to achieve the learning and classifying functions of SVM and are currently often used in a number of fields [49, 50]. The application of SVM requires training set and testing set for learning and classification, respectively. The labels for application are shown below:

(1) $$\begin{array}{c} \text{Class Label}=\left\{\begin{array}{cc} \text{Healthy}=0, & \\ \text{schizophrenia}=1. & \end{array}\right. \end{array}$$

Based on the seven eigenvalues of each person: WCST error rate, GMV, WMV, CSF volume, T1 volume, Component 11, and Component 15, as well as the defined training set label, we build an optimized predicted model and a hyperplane for classification. This predicted model was used to predict the classification of the testing set.

## 3. Results

In this study, not only the jICA was used to analyze normal and schizophrenia patients but also the brain differences based on PANSS scores were divided into two groups of schizophrenia patients. Therefore, the components of jICA, volume of brain structure, and WCST error rate were subjected to further analysis and discussion.

### 3.1. Joint ICA Results

Using weight matrix and independent joint-sources, the test group (schizophrenia) and control group (normal) data were decomposed using jICA. The result showed 15 component sources. After Bonferroni correction, *p* value should be lower than 0.05/15 = 0.003333. Only Component 15 (*p* = 0.000) and Component 11 (*p* = 0.002) were within this range. Thus, we discussed Component 15 and Component 11. The loading parameters of Component 15 and Component 11 (Figure 2) showed intergroup differences and the changes in these areas in the normal brains were more significant than those in the schizophrenia brains. Component 11 showed differences in the middle temporal gyrus, lingual gyrus, subgyral regions, cuneus, superior temporal gyrus, precuneus, superior parietal lobule, inferior parietal lobule, and middle occipital gyrus. Component 15 from the SBM analysis showed differences in the brains of schizophrenic and healthy volunteers in the middle frontal gyrus, precentral gyrus, subgyrus, postcentral gyrus, superior frontal gyrus, medial frontal gyrus, cingulate gyrus, and superior temporal gyrus.

### 3.2. Partial Correlations of WCST

We used partial correlations to control sex and age for Component 15 (all participants, only normal controls, and only patients) and WCST (percent of total error). In Figure 3, *R* square value is 0.453, and the higher loading parameters correspond to lower WCST score. When using separation analysis, *R* square value is 0.635 for normal controls only as shown in Figure 4. However, there was no significant difference in patients only between loading parameter and percentage of total error in the WCST (Figure 5).

### 3.3. Groups Based on PANSS

The patients were separated into two groups based on their positive scale (*p* scale) in PANSS, with a threshold of 24. The significant regions are the postcentral gyrus, precentral gyrus, insula, inferior parietal lobule, transverse temporal gyrus, superior temporal gyrus, and inferior semilunar lobule in the grey matter and the postcentral gyrus, inferior parietal lobule, precentral gyrus, subgyrus, middle temporal gyrus, lingual gyrus, cuneus, and middle occipital gyrus in the white matter (Figure 6).

### 3.4. Classification Results

This study obtained seven eigenvalues of participants, which included the WCST error rate, GMV, WMV, CSF volume, T1 volume, Component 11, and Component 15. All the eigenvalues were then tested by SVM algorithm, which involved random selection and 2000 tests. Five sets of tests were done to obtain an average. Datasets of training data and testing data at various proportions were used for comparison of percentages of accuracy. The result is shown in Table 2. The maximum degree of recognition reached 91.575%.

## 4. Discussion

Schizophrenia is a serious chronic mental illness. Its basic symptoms include split personality, disorganized thinking, lack of emotion, and abnormal behaviour and are characterized by the incoordination between mental activity and the environment. The diagnosis standard of schizophrenia and the actual changes in the brain have been discussed in many studies. A number of reports [51–53] have shown that the WCST performance of patients with frontal lobe impairment is significantly worse compared to those with no frontal lobe impairment. Thus, WCST is considered an effective tool in the examination of pathological changes in the frontal lobe. Recently, WCST has also been used in studying the pathophysiological basis of schizophrenia [7, 54, 55]. In addition, whether the failure in WCST of patients with schizophrenia is the same as that in patients with impaired frontal lobe has also been discussed [56–58]. Keefe [59] believed that, in order to pass the WCST examination, the patient has to possess normal memory, auditory attention, visual attention, ability to learn patterns, abstraction, classification, operating memory, and execution control that can operate a number of recognition functions simultaneously. Some studies indicated reduced grey matter in the prefrontal cortex, left orbitofrontal gyrus, left superior frontal gyrus, and bilateral medial, and middle frontal gyrus of chronic schizophrenia [60–64]. First-episode schizophrenia patients also show reduction in inferior, middle, and medial frontal and precentral gyri. In our study, Joint-Source 15 from the SBM analysis showed differences in the brains of schizophrenic and healthy volunteers in the middle frontal gyrus, precentral gyrus, subgyrus, postcentral gyrus, superior frontal gyrus, medial frontal gyrus, cingulate gyrus, and superior temporal gyrus.

**Figure 1:**
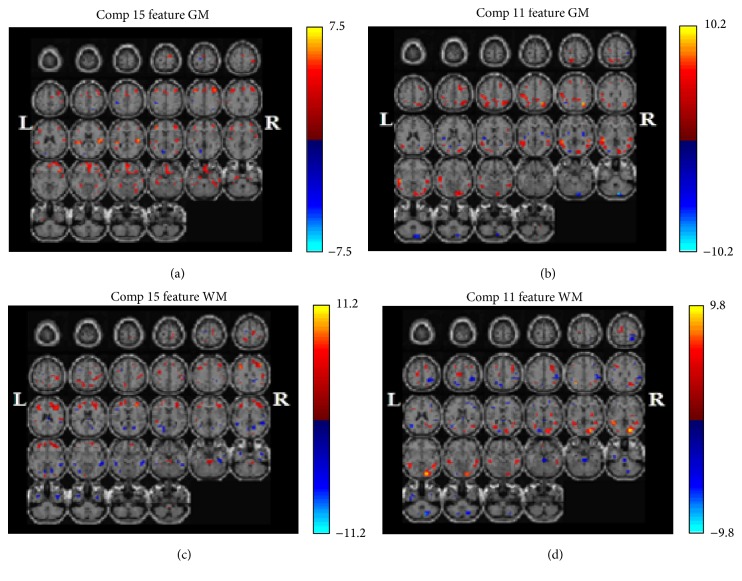
Significance maps with schizophrenia and normal. Those maps illustrating covariant decreases in the grey and white matter volume in patients with schizophrenia compared with normal controls for *p* < 0.003333: Component 15 of the grey matter (GM) (a), Component 15 of the white matter (WM) (c), Component 11 of GM (b), and Component 11 of WM (d). The left of the plane is the left side of the brain. The color represents the level of correlation: deeper red represents higher level of positive correlation, while deeper blue represents higher level of negative correlation.

**Figure 2:**
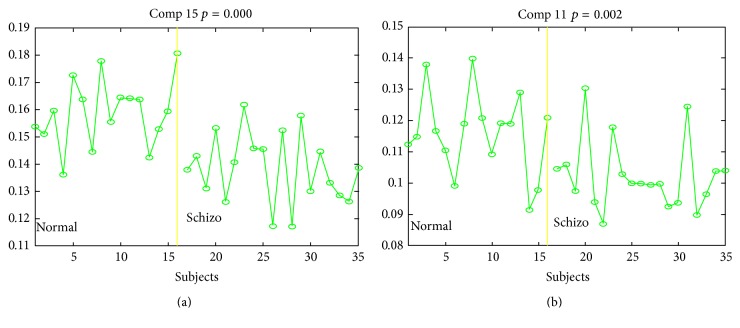
Loading parameters of Component 15 (a) and Component 11 (b). The most significant loading parameter for the *p* scale. Dividing into two groups: normal (the right of the yellow line) and schizophrenia (the left of the yellow line).

We can see significant regions in the frontal lobe, which plays a role in controlling cognition, decision, and emotion in Figure 1. When these regions are impaired, people might experience attention deficit disorders. However, lack of attention is found in schizophrenia, which would explain why we found significant changes in these regions. Some studies indicated reduced grey matter volume in the temporal lobe, particularly in the superior temporal gyrus, fusiform gyrus, and medial temporal gyrus in people with schizophrenia (chronic and first-episode schizophrenia) [65–68]. Other studies indicated grey matter reductions in the left inferior parietal gyrus in schizophrenia and bilateral postcentral gyrus in chronic and first-episode schizophrenia compared to controls [69, 70]. Tohid et al. [71] indicated moderate quality evidence suggests people with schizophrenia show reduced activity in the middle occipital gyrus during executive function tasks. In our study, Joint-Source 11 showed differences in the middle temporal gyrus, lingual gyrus, subgyral regions, cuneus, superior temporal gyrus, precuneus, superior parietal lobule, inferior parietal lobule, and middle occipital gyrus. The ICA is proved to be more consistent, therefore improving the ability to develop reliable biomarkers for disease classification.

**Figure 3:**
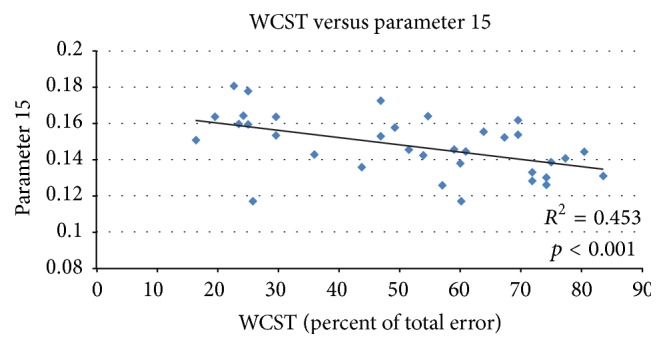
Regression analysis between loading parameters 15 and the percentages of total error on the WCST. *R* square value is 0.453; higher loading parameters correspond to lower WCST score.

**Figure 4:**
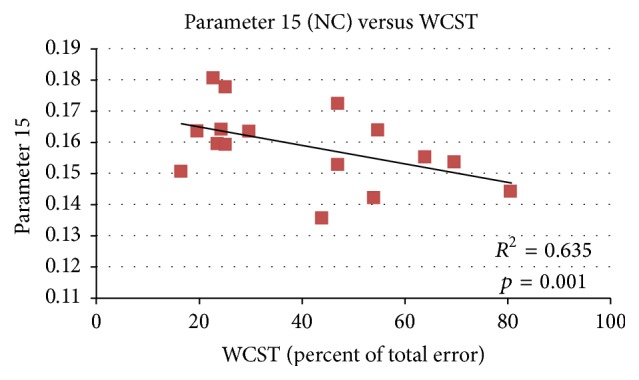
Regression analysis between loading parameters 15 (NC) and the percentages of total error on the WCST. *R* square value is 0.635; the result was more significant in normal controls than in patients.

**Figure 5:**
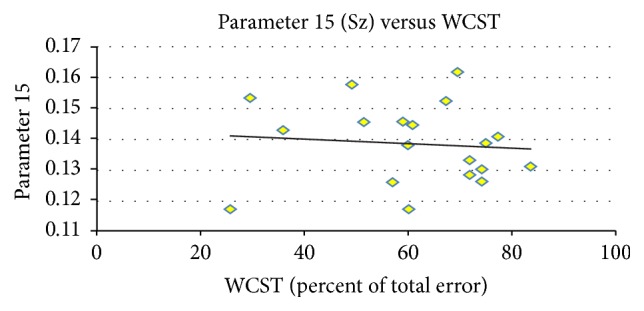
Regression analysis between loading parameters 15 (Sz) and the percentages of total error on the WCST. There is no significant difference in patients only between loading parameter and percentage of total error in the WCST.

In this research, the integration of neuroimaging data, across anatomic and functional measures, with clinical and neurobehavioural variables such as WCST and PANSS indicated the potential strength of functional studies in schizophrenia. When using partial correlations, we can see that the lower loading parameters correspond to higher percent of total error in the WCST in Figure 3. When analyzed separately, a more significant result was found in normal controls (Figure 4), and no significant finding was found in patients because of strong disease effect.

We tried to overlap the grey and white matter areas with two joint-sources, which were performed with joint ICA, and significant differences were found between patients with schizophrenia and healthy volunteers. The outcomes of this study are discussed with special emphasis on the frontal and temporal lobes. Ananth et al. figured out GMV reduction in the peristriate visual cortex (BA 18) located anteriorly to the striate visual area, which was reduced in the cohort (BA 17). Both Ananth et al. and Antonova et al. discovered the WMV reduction in the visual cortex [72, 73]. The grey and white mater volume reductions in the peristriate visual cortex (BA 18) were noted in our study. Our findings suggest that the visual cortex volume reduction is associated with a more recurrent form of the illness, since the lingual gyrus GMV was inversely correlated with the number of previous psychotic episodes.

The superior temporal gyrus GMV reduction has 100% replicability with the region of interest approach, particularly anteriorly, and voxel-based morphometry concentration studies have repeatedly observed temporal lobe reduction [72–78], which might be specific to schizophrenia since it differentiates patients with schizophrenia and those with bipolar depression.

**Figure 6:**
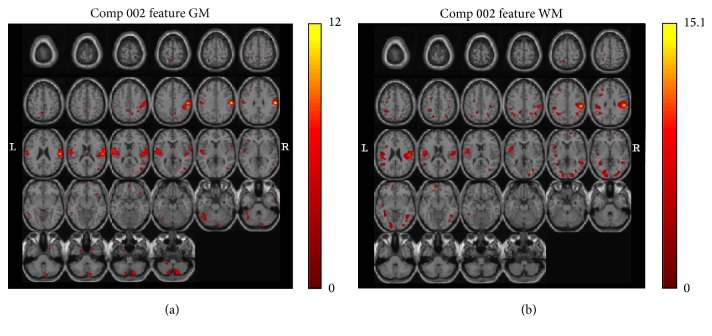
Significance maps with schizophrenia. The maps illustrating covariant decreases in the grey (a) and white matter volumes (b) in the two groups of schizophrenia based on their positive scale (*p* scale) in the positive and negative syndrome scale (PANSS), with a threshold of 24 for Component 2. The left of the plane is the left side of the brain. The color bar represents *Z*-scores.

**Figure 7:**
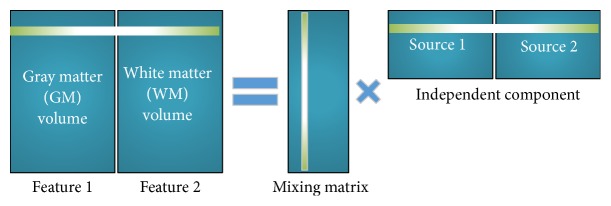
Joint ICA schematic diagram. The independent component analysis (ICA) model in which a subject in grey and white matter matrix was decomposed into mixing matrix and source matrix.

The PANSS is now well established in clinical evaluation of patients with schizophrenia. Bell et al. [79] further utilized factor analysis and separated five independent factors from the PANSS. Among them, the factors related to cognitive function originated from the difficulty in abstract thinking and rigid thinking of negative symptoms, lack of thinking organization in the active symptoms, and common mental illness pathology such as lack of judgment, impaired illness recognition, impaired focus, body tension, and weird movements. The symptom rating scales were designed to make diagnosis, categorize patients, syndromes, or both, and demonstrate antipsychotic efficacy, as well as measuring outcome. The symptom rating scales suggested limited concurrent validity with external outcome variable. In our study, we applied computerized signals from MRI and tried to correlate the relationship between patients with schizophrenia and normal control groups. In clinical practice, the positive subscale of the PANSS indicates the psychiatric symptoms of delusions, conceptual disorganization, hallucinatory behaviour, excitement, grandiosity, suspiciousness/persecution, and hostility. The higher scores (above 24) of positive subscale compared with the lower scores were further analyzed with loading parameters, which showed significant differences. In this study, *p* scale correlated with general psychopathology, suggesting the severity of illness. In the future, our efforts will be to determine how to apply the loading parameter to demonstrate the severity of illness, antipsychotic efficacy, and outcome. Finally, some parietal and temporal lobe changes were also found in only schizophrenia. *p* scale in the PANSS was a positive phenomenon score, and, hence, finding changes in the auditory cortex was reasonable in this study. Moreover, we used a *t*-test to analyze the hallucinatory behaviour on *p* scale, with the result yielding *p* = 0.059. Although *p* value is not significant enough, the result might tend to be significant if the sample size could be larger. Therefore, we think that *p* scale was controlled by the structural covariance as seen in Figure 6. The above description clearly demonstrates that the images of inner-brain structure can indicate the differences between patients with schizophrenia and normal subjects. This study also used seven eigenvalues, which included the WCST error rate, GMV, WMV, CSF volume, T1 volume, Component 11, and Component 15, for SVM learning and classification. These seven eigenvalues showed a significant proportion of the differences between patients with schizophrenia and normal subjects. Thus, this method achieved a maximum recognition rate of 91.575% and could effectively classify patients with schizophrenia and normal subjects. In addition, the SVM model built upon the result of this study also provides the possibility of research on schizophrenia prediction. Moreover, the result further indicates that the changes in the brain structure can indeed represent differences in symptoms. The implementation of such tools may be significantly beneficial in clinical interventions. People with schizophrenia may be followed up with such tools in the longitudinal follow-up study. Of course, the relatively low sample size per group limits the possibility of finding stable group differences. A more comprehensive study, with a larger sample size, is ongoing in our laboratory.

## 5. Conclusions

Our study results demonstrate that there are clear differences between the schizophrenia and normal brain structures. In addition, this study also observed that a correlation existed between the error rate of WCST and PANSS and brain structure images loaded from jICA component. The WCST is related to logic and thinking evaluation. The result showed that a higher WCST error rate represented more mistakes. The corresponding component decreased with the corresponding loading parameter, displaying reduced brain network strength and, thus, higher error rate. Apart from this, a correlation test on the WCST error rates of patients with schizophrenia and normal participants showed that normal people had higher *R* square, indicating that the reduction in this parameter resulted in more errors, while there was no significant correlation in patients only. Therefore, the correlation is more obvious in normal brains. The reason that the correlation is not so obvious in schizophrenia brains could be the strong disease effect. The PANSS is one of the important clinical parameters for schizophrenia. Schizophrenia can be classified as acute and subacute based on PANSS score, and the differences could be observed by the differences in the analysis of brain structure. Apart from statistical methods, SVM classification was also used in this study to observe the differences between schizophrenia and normal participants. The classification success rate was as high as 91.575%. The results showed that alterations in brain structure and clinical scoring could indeed effectively distinguish the two types of brains. In the future, we hope that there is great potential in the development of methods based on fMRI as a biologically based marker for medical diagnosis.

## Figures and Tables

**Table 1:** Comparison of demographic characteristics between controls and patients.

Demographic variables	Schizo (*n* = 19)	NC (*n* = 16)	*p* value
Age (years)	41.631 ± 4.94	46.88 ± 12.79	0.108
Sex (male/female)	9/10	7/9	0.922
Handedness (left/right)	0/19	2/14	0.112
PANSS P Average	21.95	0	
PANSS P Standard Deviation	6.17	0	
PANSS N Average	21.32	0	
PANSS N Standard Deviation	4.97	0	
PANSS G Average	8.52	0	
PANSS T Average	87.11	0	
PANSS T Standard Deviation	17.76	0	

**Table 2:** Percentage of accuracy test results with five sets of training data and testing data at various proportions. An average was taken from the five tests. The maximum degree of recognition was 91.575%.

	Test 1	Test 2	Test 3	Test 4	Test 5	Avg
Training data: 90% Testing data: 10%	90.625	90.775	91.425	91.575	89.875	**90.855**
Training data: 80% Testing data: 20%	90.15	89.858	89.925	89.442	89.767	**89.828**
Training data: 70% Testing data: 30%	87.861	87.633	88.35	87.878	88.106	**87.966**
Training data: 60% Testing data: 40%	85.992	85.754	85.612	85.912	85.342	**85.722**
Training data: 50% Testing data: 50%	83.079	83.291	83.291	83.185	82.794	**83.128**

## Abbreviations

MRI: Magnetic resonance imaging
WCST: Wisconsin card sorting test
PANSS: Positive and negative syndrome scale
SVM: Support vector machine
jICA: Joint independent component analysis
CSF: Cerebrospinal fluid
GMV: Grey matter volume
WMV: White matter volume
VBM: Voxel-based morphometry.
